# Supplementary material for: Identification of NAD-RNA species and ADPR-RNA decapping in Archaea
Source: Nat Commun. 2023 Nov 21;14:7597. doi: 10.1038/s41467-023-43377-x (PMC10663502; doi:10.1038/s41467-023-43377-x)
Supplement: Supplementary file 1 — Supplementary Information [file 41467_2023_43377_MOESM1_ESM.pdf]

Identification of NAD-RNA species and ADPR-RNA decapping in Archaea

José Vicente Gomes-Filho[1*], Ruth Breuer[1], Hector Gabriel Morales-Filloy[2], Nadiia Pozhydaieva[3], Andreas Borst[4], Nicole Paczia[3], Jörg Soppa[4], Katharina Höfer[3,5], Andres Jäschke[2], Lennart Randau[1,5*]

[1]Faculty of Biology, Philipps-Universität Marburg, Marburg, Germany

[2]Institute of Pharmacy and Molecular Biotechnology (IPMB), Heidelberg University, Heidelberg, Germany.

[3]Max Planck Institute for Terrestrial Microbiology, Marburg, Germany

[4]Institute for Molecular Biosciences, Biocentre, Goethe-University, Frankfurt, Germany

[5]SYNMIKRO, Center for Synthetic Microbiology, Marburg, Germany

*Corresponding authors:

José Vicente Gomes-Filho - gomesfil@staff.uni-marburg.de

Lennart Randau - lennart.randau@staff.uni-marburg.de

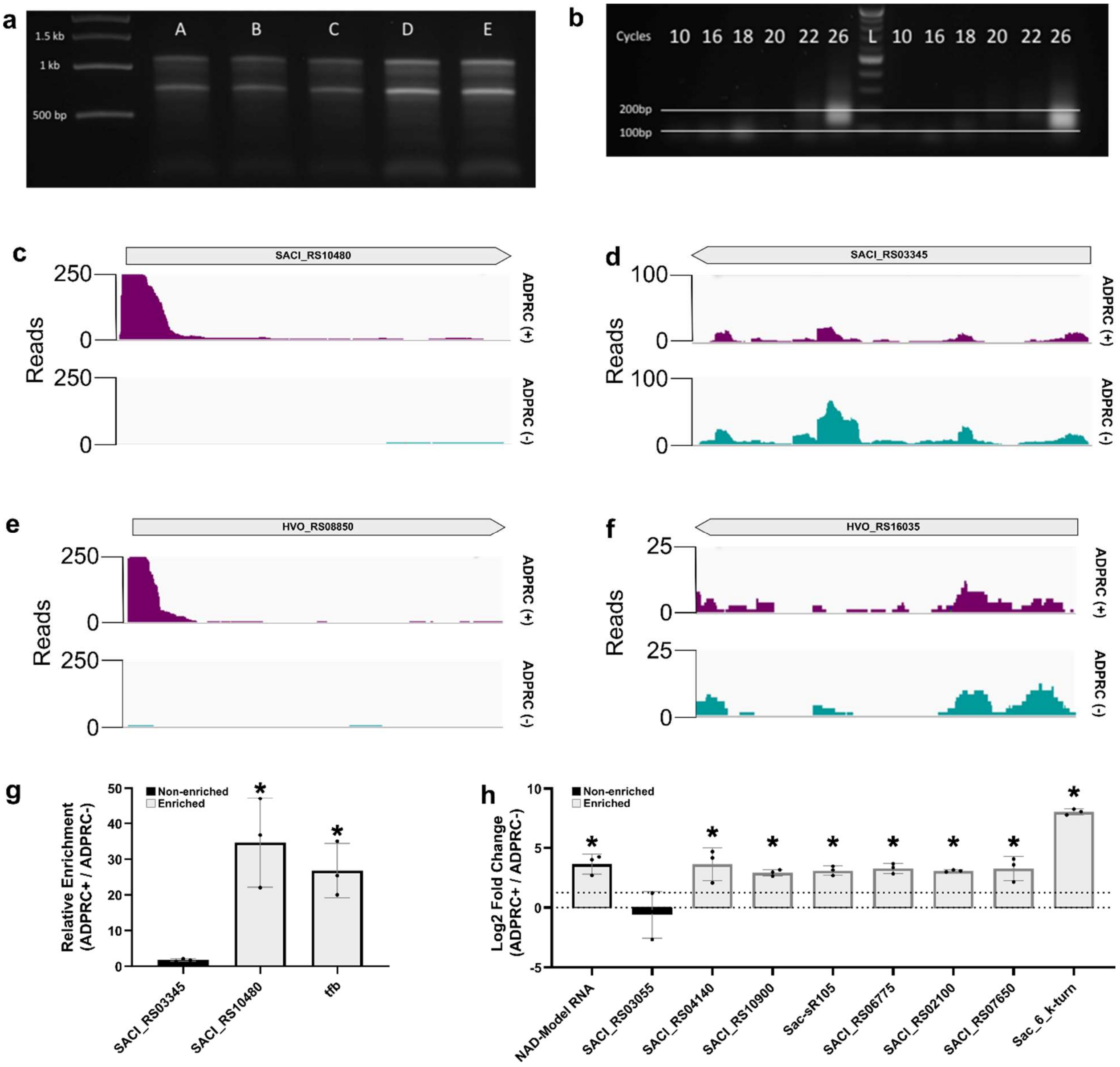

Supplementary Figure 1: a) *S. acidocaldarius* Total RNA utilized for NAD captureSeq and NAD-RNA quantification. b) Determination of the number of PCR cycles for the final amplification of NAD captureSeq libraries. The number of cycles for the final amplification was 14. c) Coverage plots of the enriched gene SACI_RS10480 from *S. acidocaldarius*. d) Coverage plots of the non-enriched gene SACI_RS03345 *S. acidocaldarius*. e) Coverage plots of the enriched gene HVO_RS08850 from *H. volcanii*. f) Coverage plots of the non-enriched gene HVO_RS16035 from *H. volcanii*. g) qPCR to validate the enrichment of specific NAD-RNAs in NAD captureSeq libraries. *tfB* and SACI_RS10480 were shown to be enriched in both experiments. SACI_RS03345 was used as a non-

enriched control. Student's t-test was used to assess significance (one-sided unpaired t-test *p=0.0033, *p=0.0043). h) Seven enriched and one non-enriched NAD-RNAs detected by NAD captureSeq were validated by ADPRC catalyzed biotinylation followed by qRT-PCR. The $2^{-\Delta\Delta Ct}$ method was used to calculate fold change enrichment (ADPRC+/ADPRC-) and student's t-test was used to assess significance (one-sided unpaired t-test, *p=0.0128, *p=0.0186, *p=0.0181, *p=0.0161, *p=0.014, *p=0.0155, *p=0.0189, *p=0.0008). Source data are provided as a Source Data file.

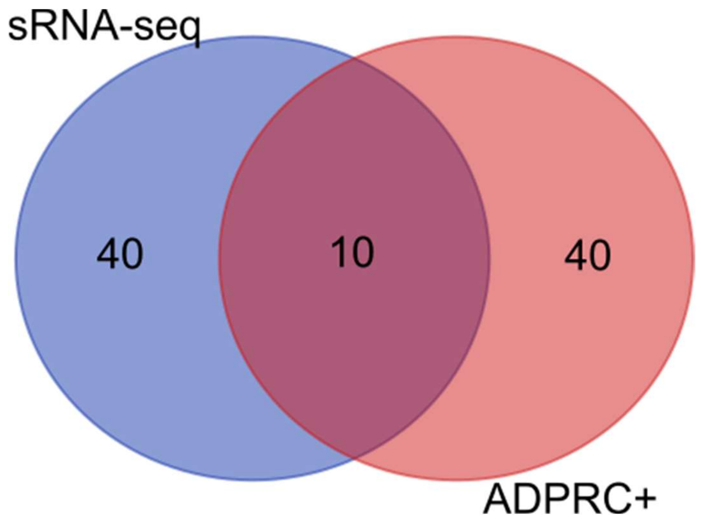

Supplementary Figure 2: Venn diagram demonstrating the overlap between the top 50 most enriched RNAs in sRNA-seq and NAD captureSeq ADPRC+ libraries (ADPRC+). Enriched NAD-RNAs are marked blue in Supplementary Table 5. It's important to note that not all ADPRC+ RNAs in this dataset are significantly enriched compared to ADPRC-samples. Source data are provided as a Source Data file.

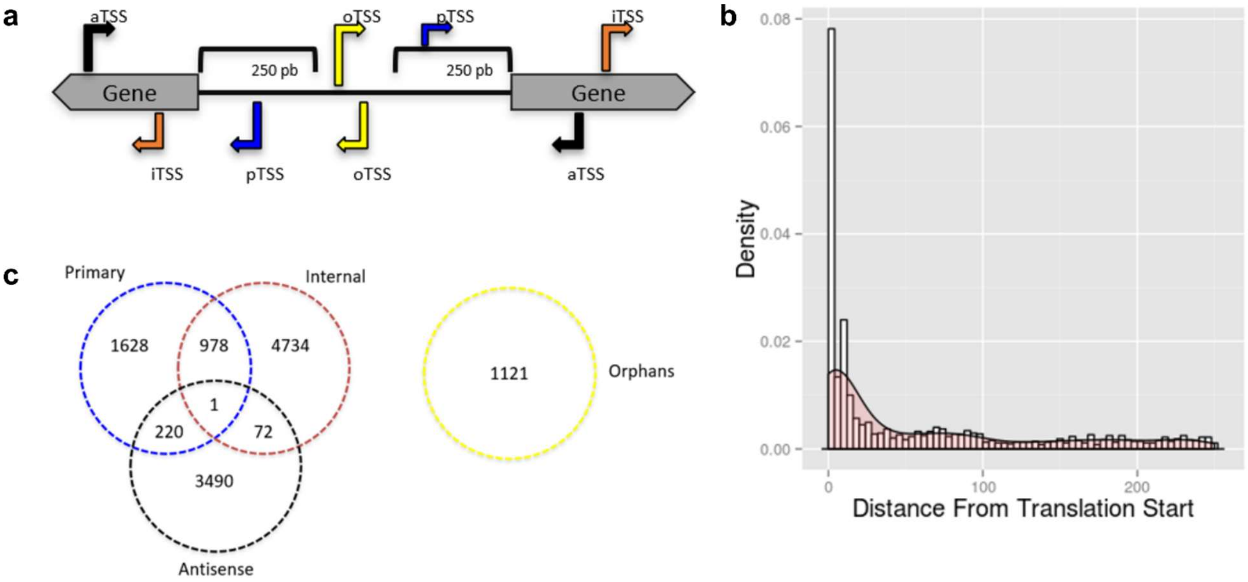

Supplementary Figure 3: dRNA-seq and transcription start site (TSS) detection in *S. acidocaldarius*. a) Classification of identified TSS according to their positions relative to neighbor genes. aTSS: antisense transcription start site; oTSS: orphan transcription start site; pTSS: primary transcription start site; iTSS: internal transcription start site. b) Distribution of distances from the translation start sites. Most TSS present a distance of 0 from the translation start site, evidencing a low number of 5´UTRs. c) TSS classification in *S. acidocaldarius*. Source data are provided as a Source Data file.

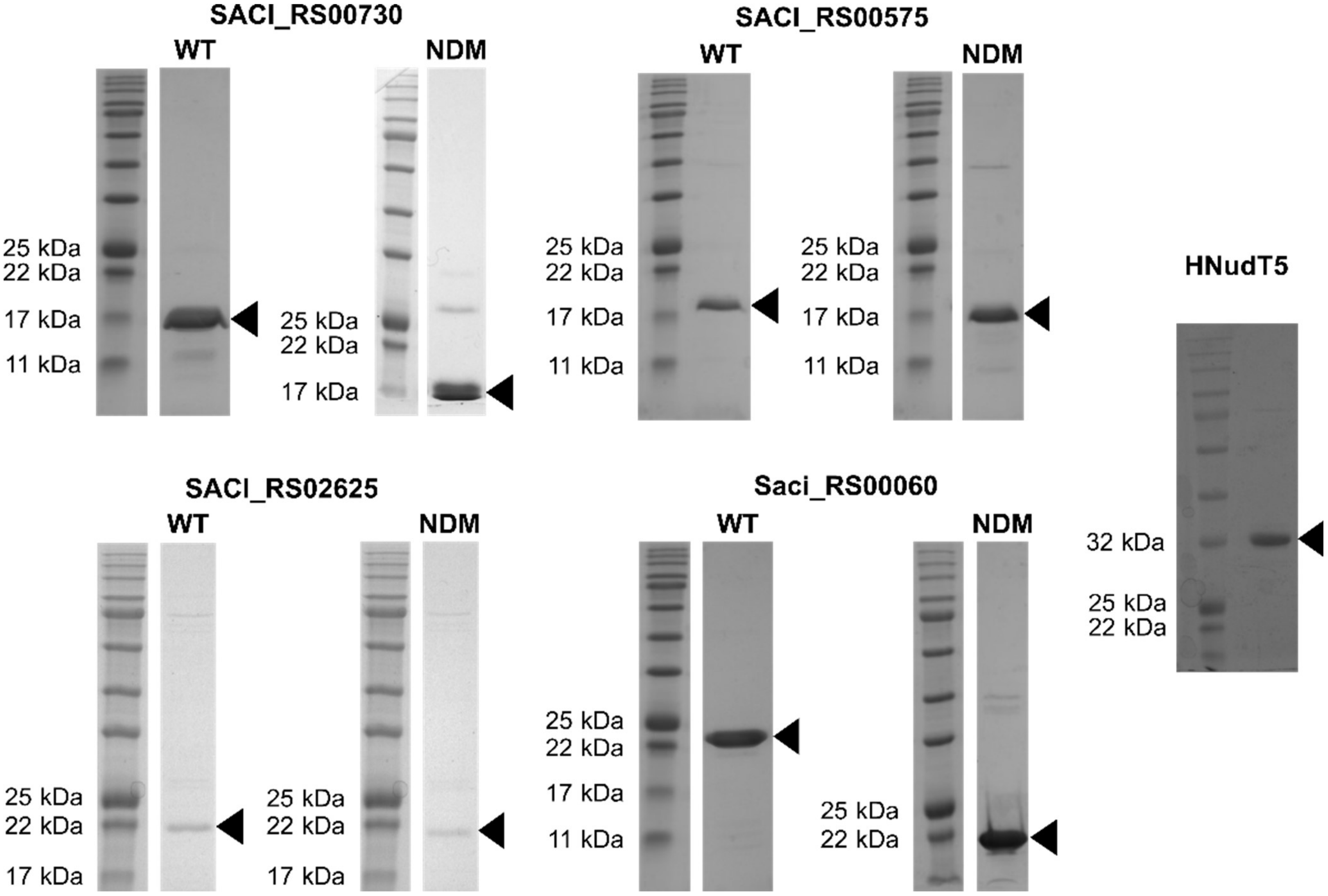

Supplementary Figure 4: SDS-PAGE of the indicated purified proteins used in this work. Source data are provided as a Source Data file.

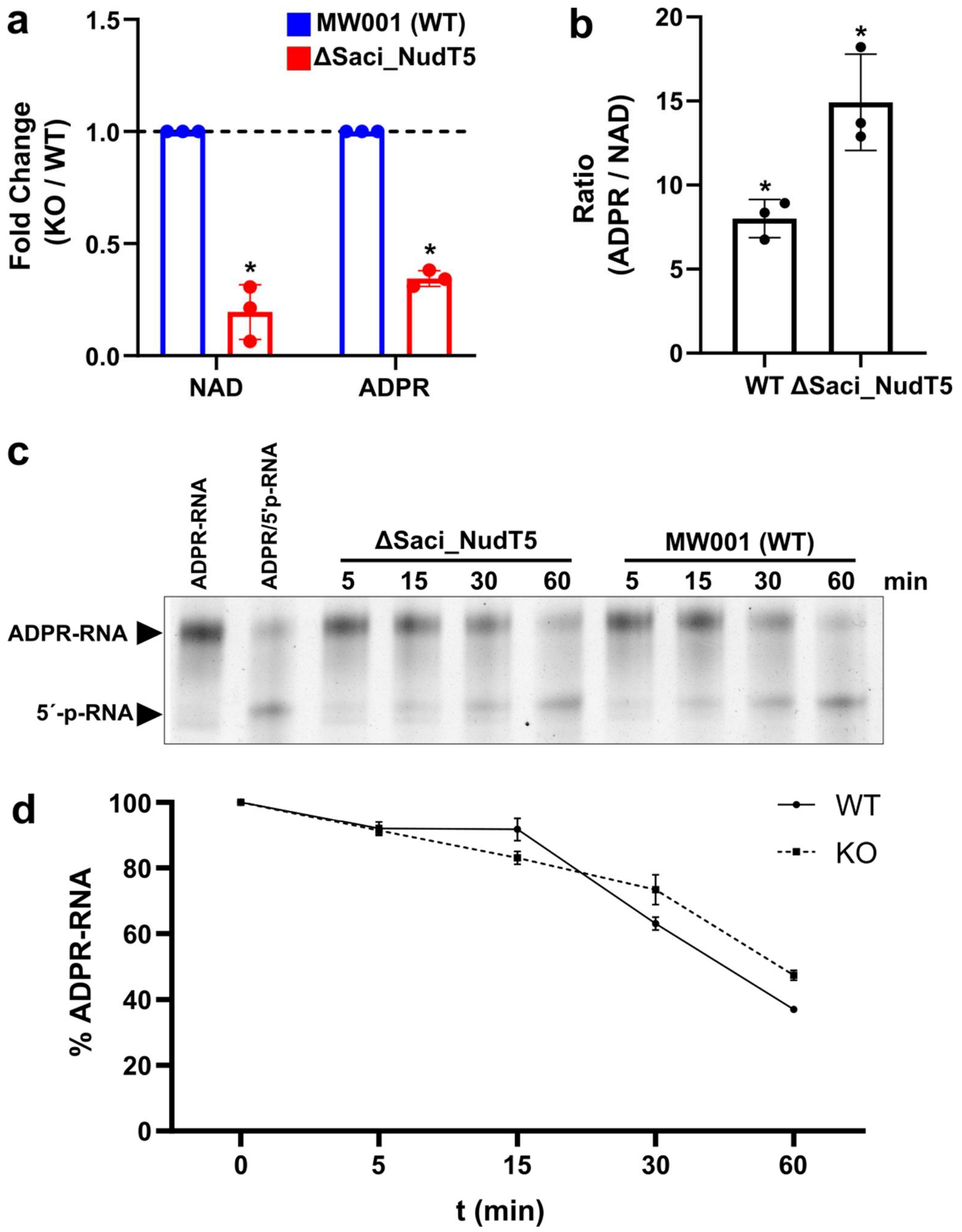

Supplementary Figure 5: Deletion of SACI_NudT5 impacts the levels of NAD- and ADPR-RNAs in *S. acidocaldarius.*

a) Ratio of NAD and ADPR in nuclease P1 digested total RNA when compared between total RNA of the WT and KO strains as determined by LC-MS/MS (Average of three independent experiments with error bars representing ±SD, two-sided unpaired t-test *p=0.0003, *p=0.0003). Dotted lines: Fold change threshold = 1. b) Ratio of ADPR over NAD in nuclease P1 digested total RNA from *S. acidocaldarius* MW001 (WT) and

ΔSACI_NudT5 (KO) determined by LC-MS/MS (Average of three independent experiments with error bars representing ±SD, two-sided unpaired t-test *p=0.037). c) Nuclease and decapping activity of S30 cell extracts (S30) from WT and KO using *in vitro* transcribed ADPR-Model RNA (38 nt). Reactions were incubated for up to 60 minutes at 65ºC, resolved on an APB polyacrylamide gels, and imaged. The image shown represents the results from one of three independently performed experiments with similar results. d) ADPR-decapping was quantified (Average of three independent experiments with error bars representing ±SD). Source data are provided as a Source Data file.

Supplementary Table 1: Concentration of NAD- and ADPR-RNAs in *S. acidocaldarius* obtained by nuclease P1 treatment of total RNA followed by LC-MS/MS analysis.

Strain	NAD (fmol / µg RNA)	ADPR (fmol / µg RNA)
S. acidocaldarius MW001	2±0.3	16±1
S. acidocaldarius ΔSaci-aCPSF2	8.7±0.6	20±1.2
S. acidocaldarius ΔSACI_NudT5	0.3±0.06	4.43±0.05

Supplementary Table 2: Potential NADases identified by HMMER using protein families listed on Supplementary Data 7.

Target Gene	old Locus	tlen	Query name	PFAM	E-Value	score	c-Evalue	i-Evalue	score	length aln	acc
**SACI_RS00210**	Saci_0047	309	IU_nuc_hydro	PF01156.22	1.20E-83	281.8	5.00E-88	1.40E-83	281.6	296	0.95
**SACI_RS03750**	Saci_0782	552	Phosphodiest	PF01663.25	2.80E-31	110	2.80E-34	7.70E-30	105.3	339	0.74
**SACI_RS05660**	Saci_1187	60	IU_nuc_hydro	PF01156.22	3.30E-12	47.3	1.20E-16	3.50E-12	47.2	50	0.93
**SACI_RS01305**	Saci_0268	238	Metallophos	PF00149.31	4.20E-11	44.2	3.60E-15	5.10E-11	44	189	0.65
**SACI_RS06380**	Saci_1335	254	Metallophos	PF00149.31	9.10E-08	33.4	7.00E-11	9.70E-07	30	112	0.72
**SACI_RS00235**	Saci_0052	60	Metallophos	PF00149.31	2.20E-06	28.9	1.60E-10	2.30E-06	28.8	41	0.94
**SACI_RS05400**	Saci_1132	60	Metallophos	PF00149.31	0.0022	19.1	1.70E-07	0.0023	19	43	0.78
**SACI_RS02045**	Saci_0419	135	Enterotoxin_a	PF01375.20	0.0055	17	2.50E-07	0.0069	16.6	67	0.88
**SACI_RS11720**	No old locus	60	Phosphodiest	PF01663.25	0.012	15.9	4.30E-07	0.012	15.8	43	0.76

Supplementary Table 3: List of oligos, plasmids, and strains used in this work.

Name	Sequence	Application
**Model-NAD-RNA-sense**	GATCACTAATACGACTCACTATTACTGTGTCG TCGTCGTCTGCTGTCTCTCTCTCGCGGGC	*In vitro* with NAD/ADPR cap
**Model-NAD-RNA-antisense**	GCCCGCGAGAGAGAGACAGCAGACGACGAC GACA CAGTAATAGTGAGTCGTATTAGTGATC	*In vitro* with NAD/ADPR cap
**RS10480-NAD-qPCR-FWD**	GATATGGGTGCTGGTGATG	NAD-seq qPCR validation / qRT-PCR from ADPRC catalyzed biotinylation of NAD-RNAS
**RS10480-NAD-qPCR-REV**	CATAACTAAGCAGGGGTGATTC	NAD-seq qPCR validation / qRT-PCR from ADPRC catalyzed biotinylation of NAD-RNAS
**RS04140-NAD-qPCR-REV_IR**	GCCGTAGAGAAAGGACTAGTCAG	NAD-seq qPCR validation / qRT-PCR from ADPRC catalyzed biotinylation of NAD-RNAS
**RS04140-NAD-qPCR-FWD_IR**	ATTCAATCCTCAATAGACAGAAAC	NAD-seq qPCR validation / qRT-PCR from ADPRC catalyzed biotinylation of NAD-RNAS
**SACI_RS03345-NAD-qPCR-REV**	TGGATGGTTAGCCATCTCAAATTG	NAD-seq qPCR validation / qRT-PCR from ADPRC catalyzed biotinylation of NAD-RNAS
**SACI_RS03345-NAD-qPCR-FWD**	AACTAAGGATAGTTGGTATCCG	NAD-seq qPCR validation / qRT-PCR from ADPRC catalyzed biotinylation of NAD-RNAS
**SACI_RS00730_HindIII**	TTTAAGCTTCTACTTGGAGGTTTGAGTGA	SACI_RS00730 with HindIII site
**SACI_RS00730_BamHI**	TTTGGATCCCATGGAACGACCTTTAGTTGC	SACI_RS00730 with BamHI site
**SACI_RS00060_HindIII**	TTTAAGCTTCTATCCTTGTAGGAGAGATC	SACI_RS00060 with HindIII site
**SACI_RS00060_BamHI**	TTTGGATCCCATGAGAATATATTCGTCTAA	SACI_RS00060 with BamHI site
**SACI_RS00575_HindIII**	TTTAAGCTTCTAGGTAAATTCGGTTAAAC	SACI_RS00575 with HindIII site
**SACI_RS00575_BamHI**	TTTGGATCCCATGGAGACATGTTTAGGAGT	SACI_RS00575 with BamHI site
**SACI_RS00730_a155c_a164c_a167c**	AGATGCTGTAAAAAGAGCAATGAAGGCGGCA ACTGCCCTAGACGTGG	triple point mutation in Nudix motif
**SACI_RS00730_a155c_a164c_a167c**	CCACGTCTAGGGCAGTTGCCGCCTTCATTGCT CTTTTTACAGCATCT	triple point mutation in Nudix motif
**SACI_RS00060_a242c_a251c_a254c**	GATCTAACTGCAAGAAGAGCGTTAGAGGCGG CAATAGGTTATGTTCCCCTT	triple point mutation in Nudix motif
**SACI_RS00060_a242c_a251c_a254c**	AAGGGGAACATAACCTATTGCCGCCTCTAACG CTCTTCTTGCAGTTAGATC	triple point mutation in Nudix motif
**SACI_RS00575_a143c_a152c_a155c**	GAATGCGTCGAAAGAGCACTTTACGCGGCGT TGGGGATCAGAGTC	triple point mutation in Nudix motif
**SACI_RS00575_a143c_a152c_a155c**	GACTCTGATCCCCAACGCCGCGTAAAGTGCT CTTTCGACGCATTC	triple point mutation in Nudix motif
**pRSF UP1**	TCTCGACGCTCTCCCTTATG	pRSFDuet-1 sequencing
**DuetDOWN1**	GATTATGCGGCCGTGTACAA	pRSFDuet-1 sequencing
**SACI_RS06775-qRT-F**	ATGGCAGATGTGAATGATTTTCTG	NAD-seq qPCR validation / qRT-PCR from ADPRC catalyzed biotinylation of NAD-RNAS
**SACI_RS06775-qRT-R**	AAGGCTGTTGTGACTTAAAGAGATTC	NAD-seq qPCR validation / qRT-PCR from ADPRC catalyzed biotinylation of NAD-RNAS
**SACI_RS03055-NAD-qPCR-REV**	ACCTTTTCTGGCATTAAAGC	NAD-seq qPCR validation / qRT-PCR from ADPRC catalyzed biotinylation of NAD-RNAS
**SACI_RS03055-NAD-qPCR-FWD**	AAGAATGAAAGGAACGCCGTC	NAD-seq qPCR validation / qRT-PCR from ADPRC catalyzed biotinylation of NAD-RNAS
**SACI_RS02100-qRT-F**	ATGCAACTTTCAAAAGGTTCTTTG	NAD-seq qPCR validation / qRT-PCR from ADPRC catalyzed biotinylation of NAD-RNAS
**SACI_RS02100-qRT-R**	GATACAACGCTTCCTAATGGTGCAG	NAD-seq qPCR validation / qRT-PCR from ADPRC catalyzed biotinylation of NAD-RNAS
**SACI_RS07650-qRT-F**	ATGACTAGTTGGGAGGAATATAAG	NAD-seq qPCR validation / qRT-PCR from ADPRC catalyzed biotinylation of NAD-RNAS
**SACI_RS07650-qRT-R**	TCAGGGTCTAGATAACCTATCTCC	NAD-seq qPCR validation / qRT-PCR from ADPRC catalyzed biotinylation of NAD-RNAS
**Sac_6_K-turn-qRT-F**	TATAAGTAGGGGTATCTAGCCCTCTG	NAD-seq qPCR validation / qRT-PCR from ADPRC catalyzed biotinylation of NAD-RNAS
**Sac_6_K-turn-qRT-R**	ATTCACGGCAGGAAGCCCTCCTC	NAD-seq qPCR validation / qRT-PCR from ADPRC catalyzed biotinylation of NAD-RNAS
**qPCR-NAD-MODEL-F**	ACTGTGTCGTCGTCGT	NAD-seq qPCR validation / qRT-PCR from ADPRC catalyzed biotinylation of NAD-RNAS

**qPCR-NAD-MODEL-R**	GCCCGCGAGAGAGAG	NAD-seq qPCR validation / qRT-PCR from ADPRC catalyzed biotinylation of NAD-RNAS
**qPCR-Sac_SR105-F**	AGGTAGTGATGAGTGCGACCG	NAD-seq qPCR validation / qRT-PCR from ADPRC catalyzed biotinylation of NAD-RNAS
**qPCR-Sac_SR105-R**	TTAAGTCAGGTTAAAGG	NAD-seq qPCR validation / qRT-PCR from ADPRC catalyzed biotinylation of NAD-RNAS
**qPCR-Sac_5s_rRNA-F**	CCACCCGGTCATAGTGAGCGG	NAD-seq qPCR validation / qRT-PCR from ADPRC catalyzed biotinylation of NAD-RNAS
**qPCR-Sac_5s_rRNA-R**	CCATCCCAGCTCAGAGAGG	NAD-seq qPCR validation / qRT-PCR from ADPRC catalyzed biotinylation of NAD-RNAS

**Construct**	**Application**	**Source**
pRSF + SACI_RS00730	Expression of SACI_RS00730 from *S. acidocaldarius*.	This study
pRSF + SACI_RS00060	Expression of SACI_RS00060 from *S. acidocaldarius*.	This study
pRSF + SACI_RS02625	Expression of SACI_RS02625 from *S. acidocaldarius*, synthesized by Genscript Inc.	This study
pRSF + SACI_RS00575	Expression of SACI_RS00575 from *S. acidocaldarius*.	This study
pRSF + SACI_RS00575_E48A_E51A_E52A	Expression of SACI_RS00575_Nudix domain mutant from *S. acidocaldarius*.	This study
pRSF + SACI_RS00730_E52A_E55A_E56A	Expression of SACI_RS00730_Nudix domain mutant from *S. acidocaldarius*.	This study
pRSF + SACI_RS00060_E81A_E84A_E85A	Expression of SACI_RS00060_Nudix domain mutant from *S. acidocaldarius*.	This study
pRSF + SACI_RS02625_E55A_E58A_E59A	Expression of SACI_RS02625_Nudix domain mutant from *S. acidocaldarius*, synthesized by Genscript Inc.	This study
	Deletion of Saci-aCPSF2 in *S. acidocaldarius*	This study
pET-28a-hNudT5	Expression of HNudT5	(Abele et al. 2020)

**Organism**	**Strains**
***E. coli* K12**	DH5α
E. coli	Rosetta 2 (DE3) pLysS
***Sulfolobus acidocaldarius* DSM639**	MW001 (Uracil Auxotroph)
***Sulfolobus acidocaldarius* DSM639**	MW001 + ∆Saci-aCPSF2
***Haloferax volcanii* H119**	Wild type

Supplementary Information Reference

1 - Abele, Florian; Höfer, Katharina; Bernhard, Patrick; Grawenhoff, Julia; Seidel, Maximilian; Krause, André et al. (2020): A Novel NAD-RNA Decapping Pathway Discovered by Synthetic Light-Up NAD-RNAs. In *Biomolecules* 10 (4). DOI: 10.3390/biom10040513.
