# Supplementary material for: Identification of NAD-RNA species and ADPR-RNA decapping in Archaea
Source: Nat Commun. 2023 Nov 21;14:7597. doi: 10.1038/s41467-023-43377-x (PMC10663502; doi:10.1038/s41467-023-43377-x)
Supplement: Supplementary file 3 — Description of Additional Supplementary Files [file 41467_2023_43377_MOESM3_ESM.pdf]

Description of Additional Supplementary Files:

Supplementary Data 1: NAD-RNAs identified by NAD captureSeq in S. acidocaldarius. DESeq2 was used to determine enriched transcripts (p-adjusted value < 0.1 and log2(Fold Change) ≥ 1) in samples treated with an ADP-ribosyl cyclase from Aplysia california (ADPRC+) versus non-treated samples (ADPRC-). Base mean: normalized mapped reads. log2FC: Log2 fold change (sample vs. negative control). P-value: Statistical significance after DESeq2 enrichment analysis. NAD-TSS: NAD transcription start sites identified by NAD captureSeq. pTSS: Primary transcription start sites identified by dRNA-seq.

Supplementary Data 2: NAD-RNAs identified by NAD captureSeq in H. volcanii. DESeq2 was used to determine enriched transcripts (padjusted value < 0.1 and log2(Fold Change) ≥ 1) in samples treated with an ADP-ribosyl cyclase from Aplysia california (ADPRC+) versus non-treated samples (ADPRC-). Base mean: normalized mapped reads. Log2FC: Log2 fold change (sample vs. negative control). P-value: Statistical significance after DESeq2 enrichment analysis. NAD-TSS: NAD transcription start sites identified by NAD captureSeq. pTSS: Primary transcription start sites identified by dRNA-seq.

Supplementary Data 3: Abundance comparison between the top 50 most represented RNAs in sRNA-seq and NAD captureSeq ADPRC+ libraries from S. acidocaldarius. NAD-capped genes are highlighted in blue.

Supplementary Data 4: Transcription Start Sites identified for S. acidocaldarius using dRNA-seq. Replicon: Accession number of the genome used for analysis. Start/Stop: TSS position. Due to size, this table is provided as an additional file.

Supplementary Data 5: Protein sequences used for NUDIX box identification.

Supplementary Data 6: Protein sequences used to search for TIR Domains and CD38 homologs in S. acidocaldarius.

Supplementary Data 7: PFAM codes utilized for the search of potential NADases in S. acidocaldarius.
