# Supplementary material for: Identification of NAD-RNA species and ADPR-RNA decapping in Archaea
Source: Nat Commun. 2023 Nov 21;14:7597. doi: 10.1038/s41467-023-43377-x (PMC10663502; doi:10.1038/s41467-023-43377-x)
Supplement: Supplementary file 9 — Reporting Summary [file 41467_2023_43377_MOESM9_ESM.pdf]

nature portfolio

Corresponding author(s): Jose Vicente Gomes Filho, Lennart Randau

Last updated by author(s): Oct 31, 2023

Reporting Summary

Nature Portfolio wishes to improve the reproducibility of the work that we publish. This form provides structure for consistency and transparency in reporting. For further information on Nature Portfolio policies, see our Editorial Policies and the Editorial Policy Checklist.

Statistics

For all statistical analyses, confirm that the following items are present in the figure legend, table legend, main text, or Methods section.

n/a	Confirmed	
[ ]	[x]	The exact sample size (*n*) for each experimental group/condition, given as a discrete number and unit of measurement
[ ]	[x]	A statement on whether measurements were taken from distinct samples or whether the same sample was measured repeatedly
[ ]	[x]	The statistical test(s) used AND whether they are one- or two-sided<br>*Only common tests should be described solely by name; describe more complex techniques in the Methods section.*
[x]	[ ]	A description of all covariates tested
[x]	[ ]	A description of any assumptions or corrections, such as tests of normality and adjustment for multiple comparisons
[ ]	[x]	A full description of the statistical parameters including central tendency (e.g. means) or other basic estimates (e.g. regression coefficient) AND variation (e.g. standard deviation) or associated estimates of uncertainty (e.g. confidence intervals)
[ ]	[x]	For null hypothesis testing, the test statistic (e.g. *F*, *t*, *r*) with confidence intervals, effect sizes, degrees of freedom and *P* value noted<br>*Give P values as exact values whenever suitable.*
[x]	[ ]	For Bayesian analysis, information on the choice of priors and Markov chain Monte Carlo settings
[x]	[ ]	For hierarchical and complex designs, identification of the appropriate level for tests and full reporting of outcomes
[x]	[ ]	Estimates of effect sizes (e.g. Cohen's *d*, Pearson's *r*), indicating how they were calculated

Our web collection on statistics for biologists contains articles on many of the points above.

Software and code

Policy information about availability of computer code

Data collection	qPCR - CFX Manager (v3.0.1224.1015)<br>MiniSeq - Local Run Manager v2<br>Gels - Insta GDS Touch 2<br>MassHunter Workstation v 10.1 (build 10.1.67)
Data analysis	Cutadapt v2.8<br>Fastqc v0.11.9<br>Hisat2 v2.2.1<br>HTSeq v2.0.2<br>DESeq2 v1.36.0<br>IGV v2.13.2<br>bedtools v2.28.0<br>samtools v1.16.1<br>Clustal Omega v1.2.4<br>Microsoft Excel Version v2210<br>R v4.2.2<br>R-Studio v2022.02.3+492<br>ggplot2 v3.4.0<br>imagej v1.53e

Inkscape v0.92
Jalview v2.11.2.5
MassHunter Quantitative Analysis v 10.2 (build 10.2.733.8)

For manuscripts utilizing custom algorithms or software that are central to the research but not yet described in published literature, software must be made available to editors and reviewers. We strongly encourage code deposition in a community repository (e.g. GitHub). See the Nature Portfolio guidelines for submitting code & software for further information.

Data

Policy information about availability of data

All manuscripts must include a data availability statement. This statement should provide the following information, where applicable:

- Accession codes, unique identifiers, or web links for publicly available datasets
- A description of any restrictions on data availability
- For clinical datasets or third party data, please ensure that the statement adheres to our policy

The generated NGS data are stored at the European Nucleotide Archive (ENA) under the project number PRJEB48624 (https://www.ebi.ac.uk/ena/browser/view/PRJEB48624). Source data for the figures, supplementary figures and information are provided as a Source Data File. Additional data are available from the corresponding authors upon request.

Research involving human participants, their data, or biological material

Policy information about studies with human participants or human data. See also policy information about sex, gender (identity/presentation), and sexual orientation and race, ethnicity and racism.

Reporting on sex and gender	n/a
Reporting on race, ethnicity, or other socially relevant groupings	n/a
Population characteristics	n/a
Recruitment	n/a
Ethics oversight	n/a

Note that full information on the approval of the study protocol must also be provided in the manuscript.

Field-specific reporting

Please select the one below that is the best fit for your research. If you are not sure, read the appropriate sections before making your selection.

[x] Life sciences [ ] Behavioural & social sciences [ ] Ecological, evolutionary & environmental sciences

For a reference copy of the document with all sections, see nature.com/documents/nr-reporting-summary-flat.pdf

Life sciences study design

All studies must disclose on these points even when the disclosure is negative.

Sample size	Experiments were performed in duplicate or triplicates, based on similar studies in the field which generated reproducible results. No statistical methods were used to determine sample size.
Data exclusions	No data were excluded.
Replication	All replication attempts were successful. The number of replicates is indicated in each figure legend.
Randomization	Randomization was not applied as this would not influence the experimental design and result interpretation. Biochemical assays and RNA-Seq approaches required defined components and conditions.
Blinding	Blinding was not suitable for this study as the biochemical experiments required knowledge of the components (i.e. in vitro transcripts, recombinant proteins and cell extracts of gene deletion strains).

Reporting for specific materials, systems and methods

We require information from authors about some types of materials, experimental systems and methods used in many studies. Here, indicate whether each material, system or method listed is relevant to your study. If you are not sure if a list item applies to your research, read the appropriate section before selecting a response.

Materials & experimental systems

n/a	Involved in the study
☒	☐ Antibodies
☒	☐ Eukaryotic cell lines
☒	☐ Palaeontology and archaeology
☒	☐ Animals and other organisms
☒	☐ Clinical data
☒	☐ Dual use research of concern
☒	☐ Plants

Methods

n/a	Involved in the study
☒	☐ ChIP-seq
☒	☐ Flow cytometry
☒	☐ MRI-based neuroimaging
